# Omics Analysis of Educated Platelets in Cancer and Benign Disease of the Pancreas

**DOI:** 10.3390/cancers13010066

**Published:** 2020-12-29

**Authors:** Giulia Mantini, Laura L. Meijer, Ilias Glogovitis, Sjors G. J. G. In ‘t Veld, Rosita Paleckyte, Mjriam Capula, Tessa Y. S. Le Large, Luca Morelli, Thang V. Pham, Sander R. Piersma, Adam E. Frampton, Connie R. Jimenez, Geert Kazemier, Danijela Koppers-Lalic, Thomas Wurdinger, Elisa Giovannetti

**Affiliations:** 1 Department of Medical Oncology, Cancer Center Amsterdam, Amsterdam UMC, VU University Medical Center (VUmc), 1081 HV Amsterdam, The Netherlands; g.mantini@amsterdamumc.nl (G.M.); l.meijer@amsterdamumc.nl (L.L.M.); rosita.paleckyte@polpharmabiologics.com (R.P.); t.lelarge@amsterdamumc.nl (T.Y.S.L.L.); t.pham@amsterdamumc.nl (T.V.P.); s.piersma@amsterdamumc.nl (S.R.P.); c.jimenez@amsterdamumc.nl (C.R.J.); 2 Fondazione Pisana per la Scienza, 56017 Pisa, Italy; m.capula@fpscience.it; 3 Department of Surgery, Cancer Center Amsterdam, Amsterdam UMC, VU University Medical Center (VUmc), 1081 HV Amsterdam, The Netherlands; g.kazemier@amsterdamumc.nl; 4 Department of Neurosurgery, Cancer Center Amsterdam, Amsterdam UMC, VU University Medical Center (VUmc), 1081 HV Amsterdam, The Netherlands; ilias@uni-plovdiv.bg (I.G.); g.intveld1@amsterdamumc.nl (S.G.J.G.I.V.); d.lalic@amsterdamumc.nl (D.K.-L.); 5 Department of Plant Physiology and Molecular Biology, University of Plovdiv, 4002 Plovdiv, Bulgaria; 6 Institute of Life Sciences, Sant’Anna School of Advanced Studies, 56127 Pisa, Italy; 7 General Surgery Unit, Department of Translational Research and New Technologies in Medicine and Surgery, University of Pisa, 56126 Pisa, Italy; luca.morelli@unipi.it; 8 Department of Clinical and Experimental Medicine, Faculty of Health and Medical Sciences, The Leggett Building, University of Surrey, Guildford GU2 7WG, UK; a.frampton@imperial.ac.uk; 9 Faculty of Health and Medical Sciences, The Leggett Building, University of Surrey, Guildford GU2 7XH, UK

**Keywords:** liquid biopsy, platelets, omics integration, regulatory mechanisms, gene expression, miRNAs, proteins, pancreatic cancer

## Abstract

**Simple Summary:**

Tumor cells are known to produce and secrete pro-coagulants that recruit blood particles such as platelets, inducing hypercoagulability. However, platelets can also influence tumor carcinogenesis and metastasis, creating a reciprocal, vicious loop with the tumors. Confrontation of platelets with tumor cells via transfer of tumor-associated biomolecules or influencing platelets biology (“education”) is an emerging concept, that has been recently proposed to create innovative platforms for biomarkers within blood-based “liquid biopsies”. In this study, we explore the intrinsic regulation and the potential “education” of platelets using -omics profiling in pancreatic cancer patients. Our results showed: (i) a high activity on RNA splicing that can lead to subsequent platelets education; (ii) enrichment of specific modified forms (isomiRs) of canonical miRNAs; and (iii) inhibition of SPARC transcription by specific class of isomiRs. Moreover, we created an interactive tool to visualize expected correlations, to facilitate further investigations on additional potential biomarkers and therapeutic tools.

**Abstract:**

Pancreatic ductal adenocarcinoma (PDAC) is traditionally associated with thrombocytosis/hypercoagulation and novel insights on platelet-PDAC “dangerous liaisons” are warranted. Here we performed an integrative omics study investigating the biological processes of mRNAs and expressed miRNAs, as well as proteins in PDAC blood platelets, using benign disease as a reference for inflammatory noise. Gene ontology mining revealed enrichment of RNA splicing, mRNA processing and translation initiation in miRNAs and proteins but depletion in RNA transcripts. Remarkably, correlation analyses revealed a negative regulation on SPARC transcription by isomiRs involved in cancer signaling, suggesting a specific ”education” in PDAC platelets. Platelets of benign patients were enriched for non-templated additions of G nucleotides (#ntaG) miRNAs, while PDAC presented length variation on 3′ (lv3p) as the most frequent modification on miRNAs. Additionally, we provided an actionable repertoire of PDAC and benign platelet-ome to be exploited for future studies. In conclusion, our data show that platelets change their biological repertoire in patients with PDAC, through dysregulation of miRNAs and splicing factors, supporting the presence of *de novo* protein machinery that can “educate” the platelet. These novel findings could be further exploited for innovative liquid biopsies platforms as well as possible therapeutic targets.

## 1. Introduction

Cancer death predictions in the US show pancreatic ductal adenocarcinoma (PDAC) expected to become the second leading cause of cancer-related deaths by 2030 [1]. The majority of PDAC patients are diagnosed late, with either locally-advanced or metastatic disease, which are typically resistant to chemotherapy. The lack of reliable biomarkers for preventive screening or early cancer detection, and the absence of effective therapies, are the main causes for the poor survival rates, ranging between 2 and 9% [2,3].

Tumor cells are known to secrete pro-coagulants or fibrinolytic substances that recruit platelets and this can induce hypercoagulability [4,5]. This is particularly common in patients suffering from PDAC, with an incidence of thrombotic complications up to 36%, and can be attributed to the procoagulant properties of PDAC cells, including the promotion of platelet activation [6,7,8].

Recent studies reported the use of platelets as an extremely promising source for cancer diagnosis and early detection biomarkers [9,10,11,12,13]. Platelets do not have a nucleus but hold a pool of megakaryocyte-derived mRNAs [14,15] and the complete machinery for *de novo* protein synthesis, resulting in dynamic modifications of protein expression [16]. Remarkably, platelets contain a vast amount of bioactive proteins which can be secreted upon activation [17]. These proteins can be either synthesized by the platelets themselves or taken up during circulation, making platelets profiling an extremely appealing tool to obtain a representative “image” of the current status of the healthy or diseased body.

It has already been shown that platelets can alter their RNA profiles when cancer cells are present, and they are referred as “tumor educated platelets” (TEPs) [18]. On this basis, previous studies generated robust classifiers to identify the cancer status based on the platelet signature in different tumor types, including PDAC [19,20,21]. However, further studies to investigate the molecular mechanisms underlying the “education” of platelets are warranted.

Evidence that platelets are capable of de novo protein synthesis [22], raised the issue of whether there is a fine-tuning of their content depending on external stimuli. During RNA splicing, intronic sequences of pre-mRNA are generally removed, while exonic sequences are joined together. However, the splicing process can create several mRNA sequences by varying the pre-RNA composition (e.g., by retaining introns or skipping exons), within a process called alternative splicing. Denis et al. identified pre-mRNA splicing as a displaced nuclear process that can occur in platelets [23]. Moreover, the presence of retained introns transcripts has been suggested to be a biologically relevant phenomenon that contribute to modulation of the platelet transcriptome [16,24].

Apart from RNA, circulating platelets are also enriched in small non-coding RNAs (ncRNAs) such as miRNAs [25,26,27]. It is well recognized that many small ncRNAs play a pivotal role in regulation of mRNAs expression in physiological and pathological conditions [28,29,30]. In particular, miRNAs bind to specific regions in the target mRNAs, thus leading to mRNA degradation or repression, subsequently resulting in suppression of protein translation [31,32,33,34].

Most miRNA genes are transcribed by RNA polymerase II in the nucleus. The pri-miRNA is then cleaved by the microprocessor Drosha. The resulting pre-miRNA is then exported by Exportin-5 in the cytoplasm where it is further cleaved by Dicer. Commonly, one strand of the miRNA is bound to AGO protein and incorporated in the RISC complex that will repress gene transcription [35]. However, malfunctions of Drosha and Dicer can create modified forms of miRNA, called “isomiRs”. Additionally, isomiRs can be generated by the addition of nucleotides to the 3′ or 5′ ends by nucleotidyltransferases such as TUT4 and GLD2. Remarkably, there are several types of isomiRs, depending on the miRNA length and the addition of nucleotides with respect to the canonical form, and they have already been described as potential biomarkers for prostate cancer detection [29].

A successful integration of data from those types of molecules (i.e., miRNAs, isomiRs, mRNA and protein expression) can lead to the discovery of new biomarkers that reflect their complex relationships as well as to understand which biological pathways are affected in different diseases, including cancer [36].

Here, to further unravel the biology underlying TEP profiles in PDAC, we applied for the first-time parallel deep omics approaches using Next Generation Sequencing (NGS) for RNA and small-RNA and label-free LC-MS/MS for proteomics, using benign pancreatic disease as ”control group” to elucidate the biology of platelets in patients affected by malignant tumor platelets.

These studies provide an extensive catalog of -omics data that can be used in many ways to explore non-coding RNAs, mRNAs and protein expression in platelets of PDAC and benign lesions. To facilitate further research on diagnostic markers from non-invasive biopsies, novel targets to inhibit metastases formation and the many further uses that can be envisaged, all data are available in GEO (GSE160252), proteomeXchange (PXD022514) and resulting biological networks from these analyses are presented in the R Shiny Web App: http://platelnet.eu.ngrok.io while the R script code is available at: https://github.com/Giulia221091/Platel-net.

## 2. Results

First, we investigated whether the inclusion of healthy donors can be useful in our study setting. We analyzed age- and sex- matched healthy donors samples (HD, *n* = 19) and their RNA expression profile when compared to PDAC and benign platelets. Based on the different RNA profile of HD (Figure S1) we have excluded this group from the following analyses. Indeed, HD cases classify aside from the other groups, suggesting that the expression of RNA from blood platelets of healthy individuals is different from the RNA expression of platelets from patients with PDAC or benign diseases. This difference might be explained by the fact that all patients with benign diseases had some inflammatory responses which could result in differences in RNA expression in platelets compared to HD.

Our choice to compare PDAC to benign disease was also sustained by the actual clinical challenge: distinguish PDAC patients from patients having non-malignant disease. Unfortunately, clinical symptoms and diagnostic features of patients with PDAC show a considerable similarity to those of patients with different benign diseases of the pancreas. Currently, the diagnostic process relies on clinical suspicion, radiological investigation, brush cytology or fine-needle aspiration for pathological confirmation, and measurement of tumor markers. However, most clinically used biomarkers fail to discriminate PDAC from benign diseases, substantiating the need for correction of the inflammatory signal in -omic analyses.

To obtain a comprehensive overview of the regulation of the transcriptome and proteome in platelets of PDAC and benign disease, we performed quantitative proteomic analysis (~2000 identified proteins), small-RNA profiling (~44,000 canonical and isomiRs type identified) and transcriptomics analysis (~50,000 RNA transcripts identified) on isolated and highly purified platelets. Platelets were collected from patients with PDAC (N = 11) and age- and sex matched patients with benign disease (*N* = 11). Clinical characteristics and age- and sex distribution are presented in Table S1 and Figure S2. In addition, proteomics analysis was performed on a subset with sufficient protein content after carefully checking that age and sex were still matching between groups in proteomics and transcriptomics datasets (Figure S2).

**Figure 1:**
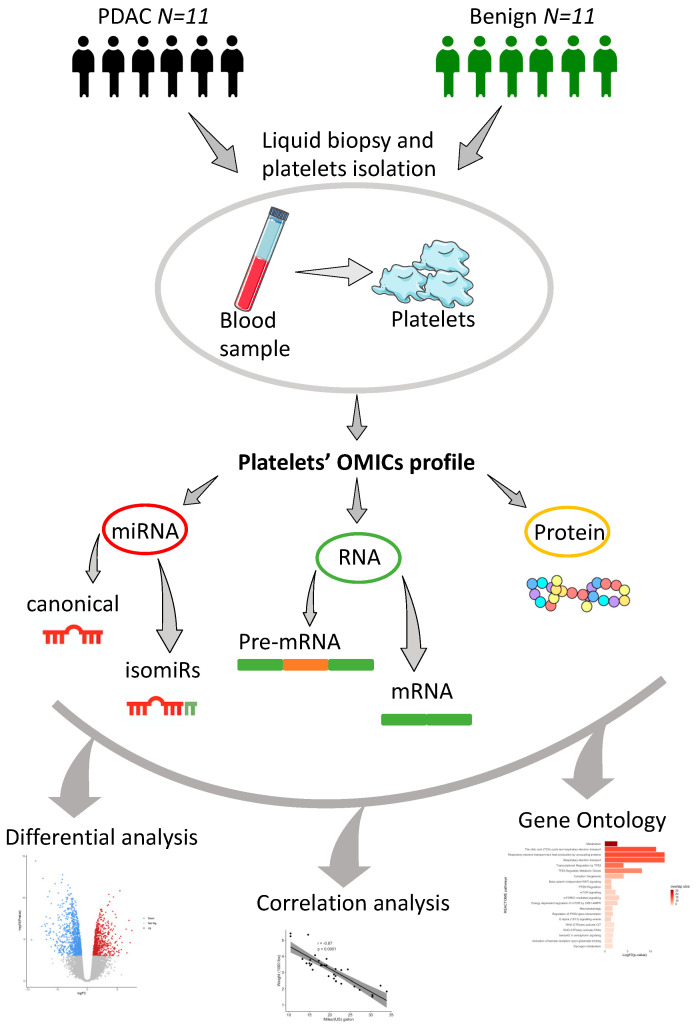
Workflow of platelets omics profiling. Platelets were isolated from 22 age and sex-matched patients with PDAC and benign diseases. Small-RNAs, RNA transcripts (PDAC samples = 11; benign samples = 11) and proteins (PDAC samples = 8; benign samples = 11) were isolated and sequenced following validated protocols [28,37]. Bioinformatics tools were adopted to quantify canonical as well as isomiRs from smallRNA-seq, mRNA and intron-spanning reads from RNA-seq, and proteins. Downstream analyses were carried out using standalone tools for differential analysis of all data types comparing PDAC versus benign platelets; intra group correlation analysis between miRNAs, mRNAs and proteins of matched PDAC and benign platelets and, lastly, gene ontology mining.

Figure 1 describes the workflow overview adopted in this study. Blood samples were collected from patients with PDAC and benign disease, with subsequent platelets isolation. After measuring and processing for small-RNAs, transcriptomics and label-free mass spectrometry-based proteomics, we evaluated differential expressed profiles for each data type. Moreover, we performed an intra group correlation analysis in PDAC and benign patients and an integrative gene ontology mining to understand the common enriched pathways between data types in PDAC and benign platelets.

**Figure 2:**
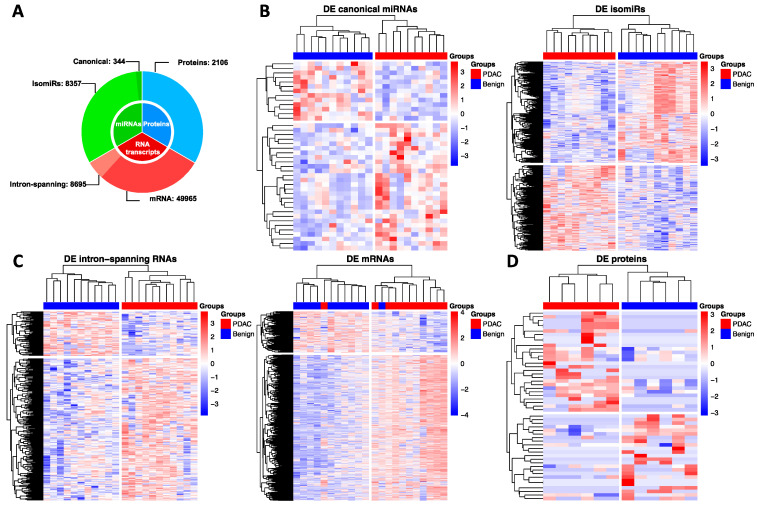
Data summary and differential analysis. (**A**) summary of canonical miRNAs, isomiRs, mRNAs, intron-spanning reads and proteins used in this study for subsequent analysis; (**B**) heatmaps of differentially expressed canonical miRNAs and isomiRs; (**C**) heatmaps of differentially expressed intron-spanning reads and mRNAs; (**D**) heatmap of differentially expressed proteins. Significance level was set to *p*-value < 0.05 and |Log_2_ FC| > 1.

A repertoire of 344 canonical miRNAs, 8357 isomiRs, 8695 intron-spanning reads, 49965 mRNAs and 2106 proteins were used for downstream analyses as reported in Figure 2A. Next, differential expression analysis was computed separately for each data-type including canonical miRNAs, isomiRs, intron-spanning reads, mRNAs and proteins (Figure 2B,C).

### 2.1. Differential Analysis

First, we aimed to differentiate platelets of PDAC and benign patients using each data-type. This analysis led to the identification of: (i) 41 differentially expressed (DE) canonical miRNAs (28 up-regulated in PDAC and 13 up-regulated in benign platelets) (Figure 2B left panel); (ii) 981 DE isomiRs (448 up-regulated in PDAC and 533 up-regulated in benign) (Figure 2B right panel); (iii) 285 DE intron-spanning reads (217 up-regulated in PDAC and 68 up-regulated in benign) (Figure 2C left panel); (iv) 1878 DE mRNAs (1466 up-regulated in PDAC and 412 up-regulated in benign) (Figure 2C right panel); (v) 52 DE proteins (26 up-regulated in PDAC and 26 in benign) (Figure 2D). Raw-sequencing data can be found at www.ncbi.nlm.nih.gov under the accession GSE160252.

Regarding the canonical miRNAs up-regulated in PDAC platelets, we observed that many of them were expected to regulate genes of ECM-receptor interaction. In particular, miR-128, miR-29a and miR-335 were the miRNAs targeting more genes involved in this interaction. However, the same miRNAs were targeting genes enriched for proteoglycans in cancer and PI3K-Akt signaling pathways. Those pathways have been described in several studies reporting their aberration in PDAC and other cancer types [38,39,40,41].

Analyzing the differentially expressed intro-spanning RNA reads we found MAP2K4, CDC42, CBL, SOS1, ROCK2, FOXO1 up-regulated in PDAC and associated to MAPK signaling, insulin signaling, TGF-beta and PDGF pathways. Previous studies already showed the alteration of those signaling pathways in PDAC cells [42,43].

However, the analysis on mature-mRNA revealed enrichment of very broad terms such as cell adhesion, cytoskeletal protein binding, and ion binding. This suggests that activated platelets in PDAC patients may require several proteins to affect different processes. Indeed, we identified many proteins up-regulated in platelets from PDAC patients that are involved in poly-A RNA binding, mRNA binding and transferase activity, suggesting a clear “protein machinery” in action [16,44].

### 2.2. Regulatory Networks

Understanding the internal regulation of platelet activity in PDAC patients is one of the main goals for this study. Thus, we used the negative correlations between miRNAs (canonical and isomiRs) and genes (intron-spanning RNAs and mRNAs), and positive correlations between genes and proteins that we call “expected” correlations. This has led to 8 resulting networks in total: four networks for PDAC platelets and four networks for benign platelets. These four networks were based on expected correlations of: (i) isomiRs-mRNA-proteins; (ii) isomiRs-intronRNA-proteins; (iii) canonical miRNA–mRNA-proteins; (iv) canonical miRNA-intronRNA-proteins.

Analyzing the resulting four correlation networks from PDAC platelets we discovered that five RNA transcripts namely SNTB1, SPARC, PPM1A, TLN1 and ADD3 were represented in all four networks. Of those transcripts, SPARC showed to be between the top five most connected nodes with a node degree ranging from three (in canonical networks) to 32 (in isomiRs networks).

**Figure 3:**
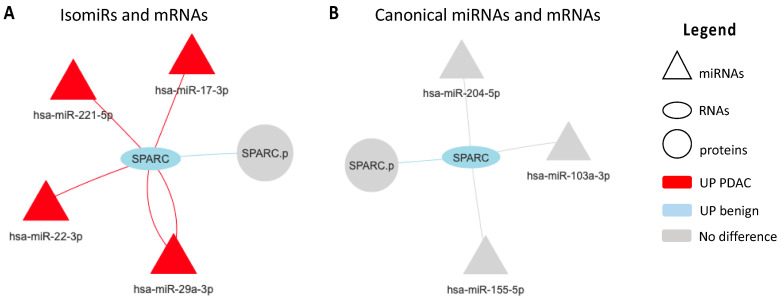
Networks focused on SPARC and based on correlation analyses of miRNAs, RNAs and proteins in PDAC platelets. A node-to-node connection is only seen if the nodes satisfy the expected correlation rule (negative correlation between miRNA and mRNAs, and positive correlation between mRNAs and proteins). (**A**) expected correlations between isomiRs and mRNA data; (**B**) expected correlations between canonical miRNAs and mRNA data; In isomiRs data each connection represents one isomiRs type, red and light blue colors represent up-regulation in PDAC and benign platelets, respectively. Triangle, oval and circle represents miRNAs, RNAs and proteins, respectively.

Specifically, the mRNA transcript of SPARC was found to be down-regulated in PDAC platelets patients. Figure 3 clearly show that SPARC down-regulation is mainly associated by isomiRs. Each connection represents one specific class of isomiR (e.g., nta#A, mv, nta#C etc.). The various number and type of isomiRs competing for SPARC down-regulation is given by the biological effects in which several miRNAs compete for the same gene target. In Figure 3A, we show the mRNA transcript SPARC is negatively correlated to several isomiRs, namely miR-17-3p, miR-29a-3p, miR-22-3p and miR-221-5p, while panel B shows canonical miRNAs associated to SPARC modulation that are not significantly different between PDAC and benign blood platelets included in our study. Of note, high expression of miR-22-3p is associated to poor survival in an external cohort of PDAC patients (Figure S3) as reported previously [45]. Moreover, KEGG pathways analysis of the above-mentioned miRNAs revealed an enrichment of classical PDAC pathways such as PI3K-Akt signaling, mTOR pathway, focal adhesion and “pancreatic cancer pathways” itself (Table S2). Additionally we have performed a multivariate analysis on SPARC and miR-29a-3p expression correcting for age, sex and stage and assessed that those clinical features are not confounding factors (Table S3).

The same analysis strategy was adopted in benign correlation networks. This time, none of the RNA transcripts was found to be over-represented between the four networks. However, we found spectrin-β non erythrocytic 1 (SPTBN1) of particular interest. This RNA transcript was one of the most connected nodes in isomiRs-networks and found to be down-regulated in benign platelets (Figure S4A,B) suggesting a key role of PDAC progression for this gene.

Previous analysis from TCGA consortium already showed a significant down-regulation of RNA transcript SPTBN1 in healthy and non-tumor matched samples when compared to PDAC tissues (Figure S4C). In addition, proteomics analysis confirmed transcriptomics results, showing elevated proteomic levels of SPTBN1 in CD24^+^ PDAC tissues when compared with CD24^−^ adjacent normal tissues [46]. However, divergent results on transcriptomics and proteomics data are found when studying prognosis of PDAC patients. Indeed, in a proteomics study of 55 pancreatic cancer patients, lower levels of SPTBN1 correlate with advanced PDAC stage and worse prognosis [47], and similar data were reported in a cohort of 82 resected PDAC patients [48] (Figure S3D). Contrary to this, transcriptomics data show that high levels of SPTBN1 correlates with poor prognosis. Therefore, different regulations at transcriptomics levels or post-translational modifications may tune SPTBN1 expression from benign state to cancer progression.

### 2.3. SPARC Is a Direct Target of miR-29a-3p and Its Modulation Affect Cell Migration

**Figure 4:**
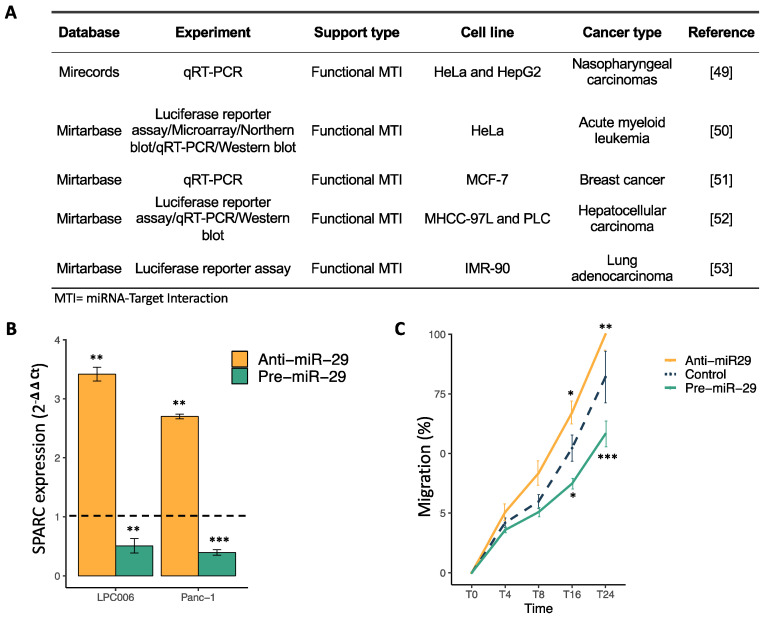
Validation of miR-29a-3p inhibitory effect on SPARC mRNA expression. (**A**) Table on previous studies validating the inhibition of SPARC caused by miR-29a-3p. (**B**) Modulation of SPARC mRNA expression levels after transfection with pre-miR-29a-3p (green) or anti-miR-29a-3p (orange) in LPC006 and Panc-1 cells. (**C**) Modulation of migration in LPC006 cells after transfection with anti-miR-29a-3p (orange), pre-miR-29a-3p (green) or miR-negative control (blue–dashed line). Significance was assessed with T-Student Test (* *p* < 0.05, ** *p* < 0.01, *** *p* < 0.0001). Dashed line refers to comparative levels in miR-negative controls.

To validate SPARC as an important target of miR-29a-3p, we evaluated previous literature studies [49,50,51,52,53] using cloned 3′UTR regions of this transcript in luciferase vectors and co-expressed them with precursor miRNAs (pre-miRs). Most of these studies showed reduced luciferase levels upon miRNA over-expression and verified a direct miRNA-mRNA interaction (Figure 4A). We then transfected both Panc-1 and LPC006 cells with the pre-miRs and anti-miRs, individually, and performed RT-qPCR to assess and confirm changes at the endogenous mRNA levels. Transfection efficiency of pre- and anti-miR-29a-3p was evaluated by qRT-PCR analysis, 48 h post transfection, showing a significant modulation of miR-29a-3p expression in both cellular models (Figure S5). Consistent with the literature findings, we observed a reduction in the levels of SPARC mRNA in cells with increased expression of miR-29a-3p. On the contrary, we observed a significant increase of SPARC expression in cells with reduced miR-29a-3p expression (Figure 4B). The effect of miR-29a-3p on cell migration was evaluated using the wound healing assay, which showed that the hsa-miR-29a-3p mimic inhibited PDAC cell migration capability, while the hsa-miR-29a-3p inhibitor enhanced it (Figure 4C). These findings demonstrate that hsa-miR-29a-3p regulates the mRNA expression of its predicted target SPARC and affects PDAC cell migration.

### 2.4. Integration of Gene Set Enrichment Analyses

Defining and discovering a regulatory mechanism in a specific phenotype is often challenging. For example, many miRNAs can compete to target the same gene and the same gene can be targeted by many miRNAs simultaneously. Not only miRNAs, but the entire family of small-RNAs can regulate gene expression by both mRNA degradation and translational repression mechanisms and it has been shown that protein expression not always correlate to gene expression due to post-translation modifications and/or regulatory feedbacks.

We hypothesized that the most active and prominent pathway that describes the current state of the cell/platelet should be maintained across all data-layers.

**Figure 5:**
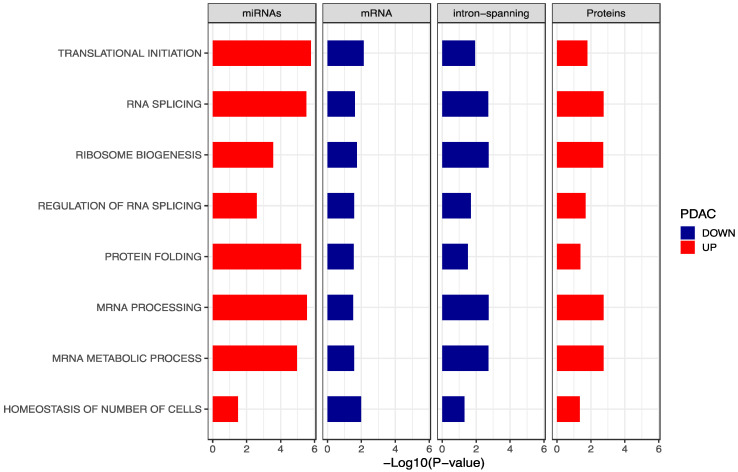
Gene ontology mining. Overlapped of significant GO terms for miRNAs, mRNAs, intron-spanning reads and proteins. Red bars describe biological terms enriched in PDAC platelets while blue bars identify biological terms down-regulated in PDAC platelets.

To this end, we performed a gene set enrichment analysis using Gene Ontology terms for each data-type. Next, results of the separate gene ontology mining were integrated retaining only the overlapped GO terms (Figure 5). Interestingly, a total of eight pathways resulted significantly enriched for RNA splicing, mRNA processing, ribosome biogenesis and translation initiation in miRNAs and proteins of PDAC platelets, in line with previous findings [16,44]. Genes and intron-spanning reads were found to be down-regulated in PDAC platelets. This can be well explained by the inhibitory mechanisms of miRNAs acting on gene expression. Of note, proteins that are supposed to correlate to RNA expression are on opposite direction. This outcome can have at least three explanations: (i) the presence of alternative regulatory mechanisms and post translational modifications on RNAs; (ii) various proteins also non-tumor related can be ingested by platelets; (iii) blood platelets can ingest miRNAs and proteins that can control internal splicing events encoding for unfolded/non-functional proteins inducing a “specific” regulation. Indeed, down-regulation of RNA splicing in platelets of cancer patients was already shown by Best et al. [19] when biologically mining the selected features for their diagnostic TEP model.

### 2.5. Different Isomirs Profile in PDAC and Benign Platelets of Patient

We performed an exploratory analysis on the different expression protein profile of eight PDAC and 11 benign platelets patients using our proteomics data and four additional datasets to validate our findings: (i) label-free quantification from DDA data of discovery cohort; (ii) label-free quantification from DIA data of discovery cohort; (iii) iq implementation of DIA data of discovery cohort [54]; (iv) published study on proteomics platelets using healthy controls and PDAC patients [9].

In this analysis, we found five highly expressed proteins in PDAC platelets: HBA1, HBD, PRDX2, CA1 and 1 down-regulated protein in PDAC platelets: AGT. Results are presented in Figure S6.

Next, we investigated whether there was a correlation with transcriptomics and small-RNAs data based on the above-mentioned analysis. Unfortunately, none of the six differentially expressed proteins were differentially regulated in transcriptomics data. However, AGT, HBD and CA1 shared a miRNA target that is up-regulated in PDAC, miR-26b-5p (Table S4).

**Figure 6:**
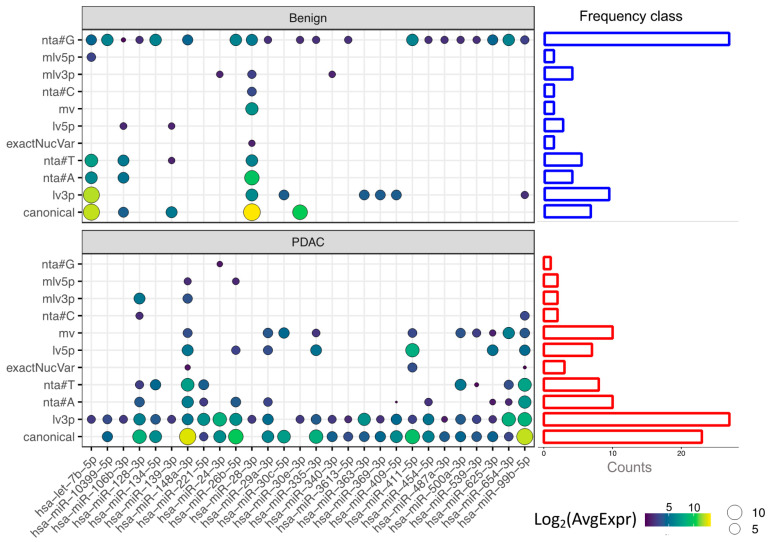
Heatmap of differentially expressed miRNAs and their average expression log_2_ transformed in benign (upper panel) and PDAC platelets (bottom panel) following the type of isomiR: (i) canonical; (ii) length variant in 3′; (iii) non-templated addition of base A; (iv) non-templated addition of base T; (v) exact nucleotide variant; (vi); length variant in 5′ (vii) multiple variant; (viii) non-templated addition of base C; (ix) multiple variant in 3′; (x) multiple variant in 5′; (xii) non-templated addition of base G. Side barplots represent the frequency of the isomiR class in benign platelets (blue) and PDAC platelets (red).

Next, we generated an isomiRs profile based on differentially expressed canonical miRNAs (Figure 6). Based on this profile, miR-26b-5p is stable in benign platelets (only addition of #G base and the 5′ ends), while several types of isomiRs of this miRNA are produced in PDAC platelets (nta#A, lv5p, lv3p, mlv5p). We then investigated the frequency of each isomiR class in PDAC and benign platelets, using only the isoform of DE canonical miRNAs (side barplots Figure 5). Strikingly, while benign platelets present a relatively high number of isomiRs of nta#G, in PDAC platelets this class of isomiR is almost absent and several different types of isomiRs appear to increase, with nta#T and lv3p being the most frequent. These results suggest a different action of nucleotidyltransferases in the two patient groups.

## 3. Discussion

This study demonstrates a novel integrative interaction of miRNAs, mRNAs, and protein expression in blood platelets of patients with PDAC, when compared to benign lesions. Parallel deep omics approaches were applied to unravel the biology of TEPs and correlate expression profiles. The intrinsic regulation of platelets was further explored using –omics profiling. With an extensive catalog of data profiles, we demonstrated profound regulations at multiple levels with high discriminatory power.

Next, correlation analysis of intron-spanning reads and mRNAs with corresponding proteins were compared in PDAC and benign platelets to evaluate the presence of different translational activity.

Importantly, SPARC resulted to be down-regulated in PDAC networks when using isomiRs data. In particular, SPARC is negatively correlated with several isomiRs, such as miR-221-5p, miR-29a-3p, miR-22-3p and miR-17-3p. We further validated the nhibitory effects of miR-29a-3p on SPARC expression through RT-qPCR in PDAC cells. Ontology mining for those miRNAs revealed an over-expression of classical PDAC pathways, such as ErbB signaling, PI3K-Akt signaling, mTOR pathway, focal adhesion. Notably, high plasma levels of miR-22 appear to be prognostic for poor survival in an external PDAC cohort [45]. However, a clear relationship between miR-22 and SPARC has not yet been described.

SPARC is a multifunctional glycoprotein with different and somehow controversial activities, it can modulate cellular interaction with the extracellular matrix (ECM) by binding collagen and vitronectin, but also contributes to counteradhesive cells effects by focal adhesion abrogation. In tumorigenesis, SPARC presents a downregulated pattern in specific tumor cell types (e.g., epithelial) and an upregulated pattern in adjacent stromal cells, as described in ovarian, pancreatic and lung cancers [55,56,57]. SPARC negatively regulates cell proliferation, angiogenesis and adhesion, but is increased in gliomas (grades II–IV) [58]. These opposing actions of SPARC may be clarified by differences in the biological activities of several proteolytic molecules including matrix metalloproteinases, cathepsins, elastases and serine proteases [59]. Moreover, SPARC regulates the activity of several growth factors such as platelet-derived growth factor, basic fibroblast growth factor and vascular endothelial growth factor that can all play a pivotal role in platelet molecular mechanisms underlying cancer progression and metastases.

In this study, SPARC negatively correlates to the above-mentioned isomiRs suggesting that alterative mechanisms such as malfunctioning on proteins involved in miRNAs biogenesis or ingestion of tumor-secreted miRNAs can potentially play a role in regulation of SPARC gene transcripts and the subsequent inhibition of the tumor suppressor. However, a still open question is if SPARC is produced by platelets and released on the tumor site, or it is taken up by platelets from tumor cells.

Finally, a possible “pathway flow” from miRNAs to proteins going across mRNAs and retained introns mRNAs was investigated. Integration of significantly enriched gene ontology terms from differentially expressed miRNAs, mRNAs, intron-spanning reads and proteins showed that RNA splicing, RNA transcription, mRNA processing and translation initiation terms were enriched in PDAC platelets for miRNAs and proteins but not for mature mRNAs and intron-spanning reads. This result is in line with previous findings [60] suggesting that other mechanisms are acting at the level of mRNA processing. For example, we found that many miRNAs related to the RNA splicing pathway, where up-regulated in PDAC platelets, whereas the mature mRNAs associated to them where down-regulated. This may suggest that other regulatory mechanisms are involved in mature-RNA degradation such as 5′ and 3′ modifications on RNA extremities [61], RNA helicases, poly-A tail elongation, chaperones and silencing RNAs (psiRNAs) [62]. However, integrated pathway analysis showed that miRNAs correlated with protein levels, meaning that, also when mRNA is degraded, proteins are still produced. A potential explanation for such phenomenon could be that miRNAs and proteins secreted by CTCs or directly from the tumor are recruited and ingested by platelets.

We demonstrated that differentially expressed canonical miRNAs of PDAC blood platelets are enriched for different type of isomiRs class such as lv3p, nta#A and nta#T while benign platelets solely show an enrichment for nta#G. There are two possible explanation for these findings: (i) those miRNAs are not produced in the platelets but can be taken up after contact with tumor, CTCs or tumor vesicles secreted; (ii) platelets contain nucleotidyltransferases that can alter the miRNA template and the subsequent interaction with the gene target. Both mechanisms can result in a modification of the platelet transcriptome and its subsequent education in response to external stimuli such as the presence of the tumor.

A seminal study performed in 2018 developed a non-invasive blood test called CancerSEEK [63] that aimed to detect eight common cancer types based on eight circulating protein biomarkers and tumor-specific mutations in circulating DNA. Depending on the cancer type, this method detected cancer with a sensitivity ranging from 69 to 98% opening a promising future in this field. More recently, an increasing number of studies has shown that cell-free DNA (cfDNA) methylation could be utilized for the identification of disease-specific signatures in pre-neoplastic lesions or chronic pancreatitis (CP), representing a sensitive and non-invasive method of early diagnosis of PDAC. An exhaustive review by Gall and colleagues reported a summary of all cfDNA studies in PDAC, chronic pancreatitis (CP) and benign lesions [64]. A major limiting factor of the reproducibility of (cf)DNA methylation data is the lack of a common reporting standard for DNA methylation detection. Furthermore, the heterogeneity of the cfDNA molecules and their genetic variability associated with cancer make the results’ interpretation even more difficult. Despite technological advances in heterogeneity deconvolution [65,66,67] pinpointing of tissue- and disease-specific subsets of molecules remains a difficult task. More insights in tumor heterogeneity will surely boost the utilization of liquid biopsies and, thus, the discovery of PDAC biomarkers.

Of note, a recent analysis reported the first methylation landscape on tumor suppressor genes in PDAC [68]. In this study higher methylation indices for SPARC were able to distinguish PDAC from CP, confirming previous observations [69]. Furthermore, SPARC hyper-methylation was associated with stage IV, metastasized disease, and poor survival. However, this study was conducted in small cohorts, and validation in larger independent cohorts is warranted.

Overall, this is the first study where miRNAs, RNAs and proteins were profiled from the same set of platelets samples in PDAC patients. Having in mind the limitation of the sample size and the low detection rate for proteomics, this study aimed to explore the intrinsic regulation of platelets using -omics profiling. To this end, we generated an extensive catalog of data profiles and an interactive tool to visualize expected correlations, in order to facilitate further investigations on additional diagnostic biomarkers and therapeutic tools.

A recent study described how tumor educated platelets enable both brain tumor diagnostic and therapy monitoring [70]. The strategy adopted behind in this study interrogated intron-spanning RNA reads and their expression using a fine-tuned classifier (swarm intelligence). This resulted in an accuracy of 0.95% when discriminating glioblastoma from asymptomatic healthy controls. Remarkably, the same method was adopted to diagnose a non-tumor disease such as multiple sclerosis with 80% of accuracy [71]. These findings demonstrate that spliced-RNA of tumor educated platelets could be a used for different diseases.

To conclude, using a multi-omics profiling of platelets from PDAC and benign diseases patients we (i) provide additional data supporting previous findings where signature of RNA splicing is down-regulated in RNA of cancer platelets; (ii) illustrated that all the -omics profiles can classify the two groups; and (iii) showed that specific isomiRs types are present in PDAC platelets prompting future studies to further validate their biological and clinical relevance.

## 4. Materials and Methods

### 4.1. Patients and Samples Collection

This study enrolled 11 patients with pathologically confirmed PDAC and 11 patients with benign disease, both groups comprising male and female subjects aged between 39 and 81 years. Clinical characteristics of the study subjects are listed in Table S1.

Blood samples were obtained from the two University Hospitals of Amsterdam (VUmc and AMC, Amsterdam Universities Medical Centers (Amsterdam UMC, Amsterdam, The Netherlands), after receiving an informed consent of the patients (medical ethical approved protocol: #14438). Healthy donors were reported to be without any type of cancer, currently or in the past, as described previously [72]. The samples and associated clinical data of all individuals was collected and stored with a retraceable code, and fully anonymized.

### 4.2. Isolation of RNA and Protein for miRNAs, mRNAs and Protein Profiling

To elucidate potential different regulatory mechanisms, miRNA, mRNA and proteins were profiled from the same blood platelets samples.

Platelet pellets were isolated within 48 h after blood collection and stored in RNAlater at −80°. Total RNA was extracted from platelets using the MiRVAna kit (Ambion) while the protein fraction was stored for proteomics analysis after removal of RNA. Platelets isolation and extraction was performed as described previously [37].

#### 4.2.1. RNA-Seq Library Preparation

For RNA isolation and sequencing, all samples were subjected to the thromboSeq protocol described previously [37].

#### 4.2.2. miRNA-Seq Library Preparation

Preparation of miRNA libraries and sequencing was performed as described by Koppers-Lalic et al. [28,29]. After RNA isolation, samples were adjusted to have the same amount of total RNA concentration (500 pg in 7 μL). Libraries were prepared using the standard small-RNA Library Prep Kit for Illumina. To assess samples’ quality before sequencing, the concentration of the samples was determined using the Fragment Analyzer and all samples met the quality requirements. Sequencing was performed using the HiSeq 4000 instrument (Illumina, San Diego, CA, USA) with 150 bp paired-ends.

#### 4.2.3. Protein Extraction and MS/MS Sample Preparation

After RNA removal proteins were precipitated and loaded on SDS-PAGE. Image J analysis was used to enable equal loading on a subsequent SDS-PAGE (12%). Proteins were allowed to run just into the running gel before the voltage was stopped and stained with Coomassie R-250. After washing in MQ, each stained protein blob was cut from the gel as a single band and subjected to tryptic (Promega, Madison WI, USA) digestion. Peptides were extracted, desalted and dried.

Peptides were separated by an Ultimate 3000 nanoLC system (Dionex LC-Packings, Amsterdam, The Netherlands). After injection, peptides were trapped at 6 μL/min on a 10 mm × 100 μm ID trap column packed with 5 μm 120 Å ReproSil Pur C18 aqua at 2% buffer B (buffer A: 0.5% acetic acid in ultrapure water; buffer B: 80% ACN + 0.5% acetic acid in ultrapure water) and separated at 300 nL/min in a 10–40% buffer B linear gradient in 90 min (120 min inject-to-inject).

Eluting peptides were ionized into a Q Exactive mass spectrometer (Thermo Fisher, Bremen, Germany). Intact masses were measured at resolution 70.000 (at *m/z* 200) in the orbitrap using an AGC target value of 3E6 charges. The top 10 peptide signals (charge-states 2+ and higher) were submitted to MS/MS in the HCD (higher-energy collision) cell (1.6 amu isolation width, 25% normalized collision energy). MS/MS spectra were acquired at resolution 17.500 (at *m/z* 200) in the orbitrap using an AGC target value of 1E6 charges, a maxIT of 60 ms and an underfill ratio of 0.1%. Dynamic exclusion was applied with a repeat count of 1 and an exclusion time of 30 s. More details on the sample processing and for DIA method are reported in the metadata file of PXD with accession: PXD022514.

### 4.3. Downstream Analysis of miRNAs, mRNAs and Protein Profiles

#### 4.3.1. Intron-RNA Processing Data

FASTQ files obtained from RNA-seq experiment were subjected to a standard pipeline as described previously [60], selecting only the spliced intron-spanning RNA reads. Gene counts were converted to counts per millions (CPM) before filtering procedures. Genes with a total count of at least 1 CPM in more than 40% of the samples were retained. Black and white cases were also retained allowing 0 values in max 18% of the positive samples group. Gene counts were then converted to TMM values through the EdgeR package in R (version 3.5.0). Differential analysis was performed with EdgeR package in R (version 3.5.0).

#### 4.3.2. mRNA Processing Data

FASTQ files were checked for quality reads and adapters were removed with Trimmomatic [73]. Successfully quality passed reads were mapped to the human reference genome (Hg19) with STAR mapping tool [74] and gene counts were extracted with HTSeq [75]. Normalization, quality filtering and data presence were previously described in Section 4.3.1.

#### 4.3.3. miRNA Processing Data

cDNA libraries were sequenced at GenomeScan (Leiden, The Netherlands) and FASTQ files were acquired. Trimming, mapping and miRNA counts was performed with sRNAbench tool (version 10/14) [76]. Profiled miRNAs were then converted to CPM following the same filtering and normalization procedure for mRNA data processing. Differential analysis was performed with EdgeR package in R (version 3.5.0).

#### 4.3.4. Protein Processing Data

MS/MS spectra were searched against the Swissprot FASTA file (release September 2015, 20197 entries, canonical and isoforms) using MaxQuant version 1.5.2.8. Peptide and protein identifications were filtered at an FDR of 1% using the decoy database strategy. The minimal peptide length was seven amino-acids. Proteins that could not be differentiated based on MS/MS spectra alone were grouped to protein groups (default MaxQuant settings). Searches were performed with the label-free quantification option selected. After contaminants removal proteins were normalized by global median. Differential analysis of spectral counts was computed in R with “ibb” package [77]. Normalized spectral counts data are reported in Table S5. Details in the data processing protocols and DIA samples are reported in the metadata file in PXD with accession PXD022514.

### 4.4. Differential Expression Analysis

In this study, two different statistical tests were adopted to determine the difference in the expression profiles of platelets in PDAC patients and patients with benign diseases. Differentially expressed canonical and isoforms of miRNAs were analyzed separately with the R package EdgeR (version 3.24.3) using cutoff of *p*-value < 0.05 and |Log2FC| > 1. Same parameters were applied for mRNAs and pre-mRNAs differential expression analysis. Finally, differentially expressed proteins were evaluated with the R package “ibb” and significance was determined using a threshold of *p*-value < 0.05.

### 4.5. Correlation Analysis

Correlation analyses were computed in R version 3.5.1. with cor.test() function. Significance level was set to *p*-value < 0.05.

### 4.6. Cell Culture and Transfection

Panc-1 cells were purchased from the American Type Culture Collection (ATCC, Manassas, VA, USA), while the primary cells LPC006 were isolated from laser-microdissected PDACs, as described previously [78]. Both were maintained in RPMI supplemented with 10% FCS, 1% penicillin/streptomycin, and 1% glutamine, at 37 °C under an atmosphere of 5% CO_2_ in 75 cm^2^ tissue culture flasks (Greiner Bio-One GmbH, Frickenhausen, Germany). When the cells were ready for transfection, they were plated the day before and then transfected with the precursor and antisense oligonucleotides (Pre-miR™ miRNA Precursor pre-miR-29a-3p and Anti-miR™ miRNA Inhibitor anti-miR-29a-3p) purchased from ThermoScientific/Ambion-Applied Biosystems, Waltham, MA, USA (Assay ID, PM12499 and AM12499, respectively) at 30 nM final concentration, as described previously [79]. Cells were plated at 5000 cells/well in 200 µL RPMI with 10% FBS and 1% antibiotics. After 24 h cells were exposed to 0.9 µL oligofectamine (Invitrogen, Paisley, UK) in serum-free medium, mixed for 10 min at room temperature, followed by the addition of 0.3 µL of 6.25 µM miR-29a-3p precursor or inhibitor. Cells were also incubated with miRNA negative controls. After overnight exposure the medium was removed from the wells and replaced with RPMI with 10% FBS, without antibiotics. Then cells were allowed to grow for additional 48 h in drug-free medium before lysis and RNA extraction to evaluate the transfection efficiency and modulation of SPARC mRNA level and cell migration.

### 4.7. Quantitative Real-Time PCR (RT-qPCR)

Total RNA extracted was used to perform RT-qPCR using Taqman mature miRNA primers and probes. RNA was extracted using a Trizol-chloroform protocol (Sigma, St. Louis, MO, USA). RNA yields and purity were checked by measuring optical density at 260/280nm with a Nanodrop^®^ spectrophotometer (NanoDrop-Technologies, Wilmington, DE, USA). Briefly, both mature miRNA miR-29a-3p and SPARC mRNA expression were measured using specific primers (assay-ID 002112 and Hs00234160_m1, respectively) for complementary DNA (cDNA) synthesis followed by Taqman PCR analysis. PCR reactions were performed on a 7500HT sequence detection system (Applied Biosystems, Foster City, CA, USA), in accordance with the manufacturer’s instructions. Duplicate samples and endogenous controls for miRNA and mRNA normalization (snRNA U6 and GAPDH) were used throughout. Quantification of miRNA relative expression was performed evaluating the threshold cycle (Ct) and normalized to U6, as described previously [79]. Similarly, expression levels of SPARC mRNA were normalized to GAPDH, and quantitation of gene expression was performed using the ΔΔCt calculation, where the amount of target gene, normalized to GAPDH and relative to the calibrator (untreated control cells), is given as 2^−ΔΔCt^. Specimens were amplified in triplicate with appropriate non-template controls, and the coefficient of variation was <1% for all replicates.

### 4.8. Migration Assay

For the migration assay the cells transfected with pre-miR-29a-3p or anti-miR-29a-3p or miR-negative control, as described above, were plated at a density of 2 × 10^4^ cells/well onto 96 wells plates, and artificial wound tracks were created by scraping with a specific scratcher within the confluent monolayers. After removal of the detached cells by PBS washing, the medium was refreshed and cells ability to migrate into the wound area was assessed by comparing the pixels of the wound tracks in the images taken at the beginning of the exposure (time 0), with those taken after 8, 16, and 24 h, with the LeicaDMI300B-station integrated with Scratch Assay software (Digital-Cell Imaging Labs, Keerbergen, Belgium), as described previously [80].

### 4.9. Functional Pathways Enrichment

To investigate the regulatory role of miRNA in PDAC and benign disease, a GSEA was performed with fgsea R package (version 1.8.0). Differentially expressed intron-spanning reads and proteins were ranked based on their *p*-value and FC [−log10(*p*-value)*FC sign]. A compendium of Gene Ontology terms (biological processes, molecular function and cellular component) was downloaded from the Broad Institute: MSigDB [81] version 6.0, including Hallmarks of Cancer.

MiEAA web based tool [82] was used to evaluate biological processes enrichments in miRNAs. Performing an over-representation analysis, the normalized enrichment score (NES) was not given by the database and was set to 1.5 for each significant enrichment.

## 5. Conclusions

In the present study, we investigated the biological mechanisms acting in platelets of PDAC patients. Patients with benign disease were included in this study as a reference for inflammatory noise. Through an extensive gene ontology mining of different –omics data we demonstrated that active RNA processing, splicing signals events and translation initiation are specific terms in the biology of circulating platelets in patients with PDAC. In particular, the differential regulation of some genes, such as SPARC, in PDAC and benign platelets, makes platelets an interesting source for diagnostic tools, together with the different miRNAs and proteomic profiles. Further understanding of the real function of these profiles is essential for biomarker discovering when using a machine learning approach. The analysis of specific platelets content could therefore provide a dynamic and powerful approach for the specific diagnosis of PDAC, paving the way for early detection intervention strategies, which represent the greatest hope for making substantial improvements in survival for this disease.

## Data Availability

The data presented in this study are available in GEO repository under ID accession: GSE160252 (raw RNA-seq data); proteomeXchange under ID accession: PXD022514 (raw proteomics data); biological networks are presented here: http://platelnet.eu.ngrok.io and the R script code is available at: https://github.com/Giulia221091/Platel-net.

## Supplementary Materials

The following are available online at https://www.mdpi.com/2072-6694/13/1/66/s1, Figure S1. Clustering of Healthy donors (HD). (a) Age distribution of benign, HD and PDAC patients included in the analysis. (b) clustering of differentially expressed intron-spanning reads between HD, PDAC and benign patients. Dashed line underlines the separation between the two main clusters. Figure S2. Age-gender match for transcriptomics and proteomics data in platelets of PDAC and benign patients. Figure S3. KM curve on plasma levels of miR-22 in PDAC patients. Figure S4. A. expected correlations with isomiRs and intron-spanning reads focused on SPTBN1. B. expected correlations with isomiRs and mRNAs focused on SPTBN1. C. differential analysis of SPTBN1 on RNA-seq data on PAAD-TCGA tissues and normal matched samples. D. Immunohistochemistry validation of SPTBN1 in resected pancreatic cancer patients. Figure S5. Modulation of miR-29a-3p levels reflecting the transfection efficiency of pre- and anti-miR-29a-3p, as assessed by qRT-PCR, 48 h post transfection. Significance was assessed with T-Student Test (* *p* < 0.05, ** *p* < 0.01, *** *p* < 0.0001). Dashed line refers to comparative levels in miR-negative controls. Figure S6. In silico validation of protein biomarkers for PDAC diagnosis from blood platelets. Table S1. Clinicopathological characteristics of PDAC and benign patients included in this study. Table S2. KEGG analysis on SPARC-MiRNA targets for intron-spanning reads and mRNAs. Table S3. Multivariate regression of clinical features associated to SPARC and miR-29 expression. Table S4. Putative protein biomarkers resulting from proteomics analysis. Table S5. Normalized spectral counts data of matched proteomics samples.

## Abbreviations

lv3p	length variant in 3′
lv5p	length variant in 5′
nta#A	non-templated addition of base A
nta#T	non-templated addition of base T
nta#C	non-templated addition of base C
nta#G	non-templated addition of base G
exactNucVar	exact nucleotide variant
mv	multiple variant
mlv3p	multiple length variant in 3′
mlv5p	multiple length variant in 5′
