# Supplementary material for: Omics Analysis of Educated Platelets in Cancer and Benign Disease of the Pancreas
Source: Cancers (Basel). 2020 Dec 29;13(1):66. doi: 10.3390/cancers13010066 (PMC7795159; doi:10.3390/cancers13010066)
Supplement: Supplementary file 1 [file cancers-13-00066-s001.zip › suppl.Figures/TableS3.pdf]

	N	*P*-value (SPARC)	*P*-value (miR-29)
**Age**			
Low (<65)	9	0.88	0.28
High (>65)	13		
**Sex**			
Male	7	0.06	0.11
Female	15		
**Stage**			
I-IIA	5	0.59	0.11
IIB	6		
