# Supplementary material for: Omics Analysis of Educated Platelets in Cancer and Benign Disease of the Pancreas
Source: Cancers (Basel). 2020 Dec 29;13(1):66. doi: 10.3390/cancers13010066 (PMC7795159; doi:10.3390/cancers13010066)
Supplement: Supplementary file 1 [file cancers-13-00066-s001.zip › suppl.Figures/FigureS4.pdf]

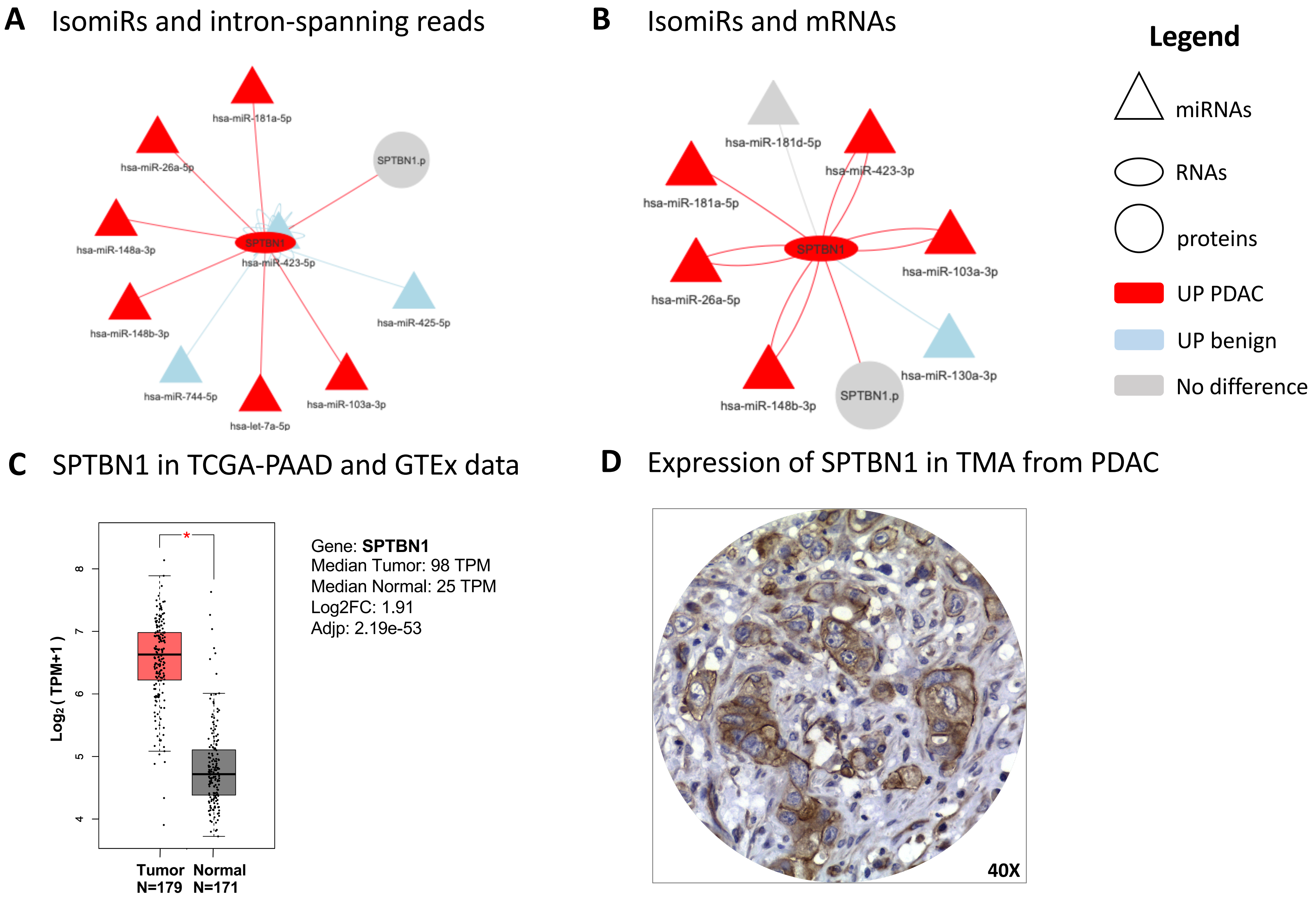

A IsomiRs and intron-spanning reads
hsa-miR-181a-5p
hsa-miR-26a-5p
SPTBN1.p
hsa-miR-148a-3p
SPTBN1
hsa-miR-423-5p
hsa-miR-425-5p
hsa-miR-148b-3p
hsa-miR-744-5p
hsa-let-7a-5p
hsa-miR-103a-3p
B IsomiRs and mRNAs
hsa-miR-181d-5p
hsa-miR-423-3p
hsa-miR-181a-5p
SPTBN1
hsa-miR-26a-5p
hsa-miR-103a-3p
hsa-miR-130a-3p
hsa-miR-148b-3p
SPTBN1.p
Legend
miRNAs
RNAs
proteins
UP PDAC
UP benign
No difference
C SPTBN1 in TCGA-PAAD and GTEx data
Log2 (TPM+1)
Tumor N=179
Normal N=171
Gene: SPTBN1
Median Tumor: 98 TPM
Median Normal: 25 TPM
Log2FC: 1.91
Adjp: 2.19e-53
D Expression of SPTBN1 in TMA from PDAC
40X
