# Supplementary figures and images for: Omics Analysis of Educated Platelets in Cancer and Benign Disease of the Pancreas
Source: Cancers (Basel). 2020 Dec 29;13(1):66. doi: 10.3390/cancers13010066 (PMC7795159; doi:10.3390/cancers13010066)

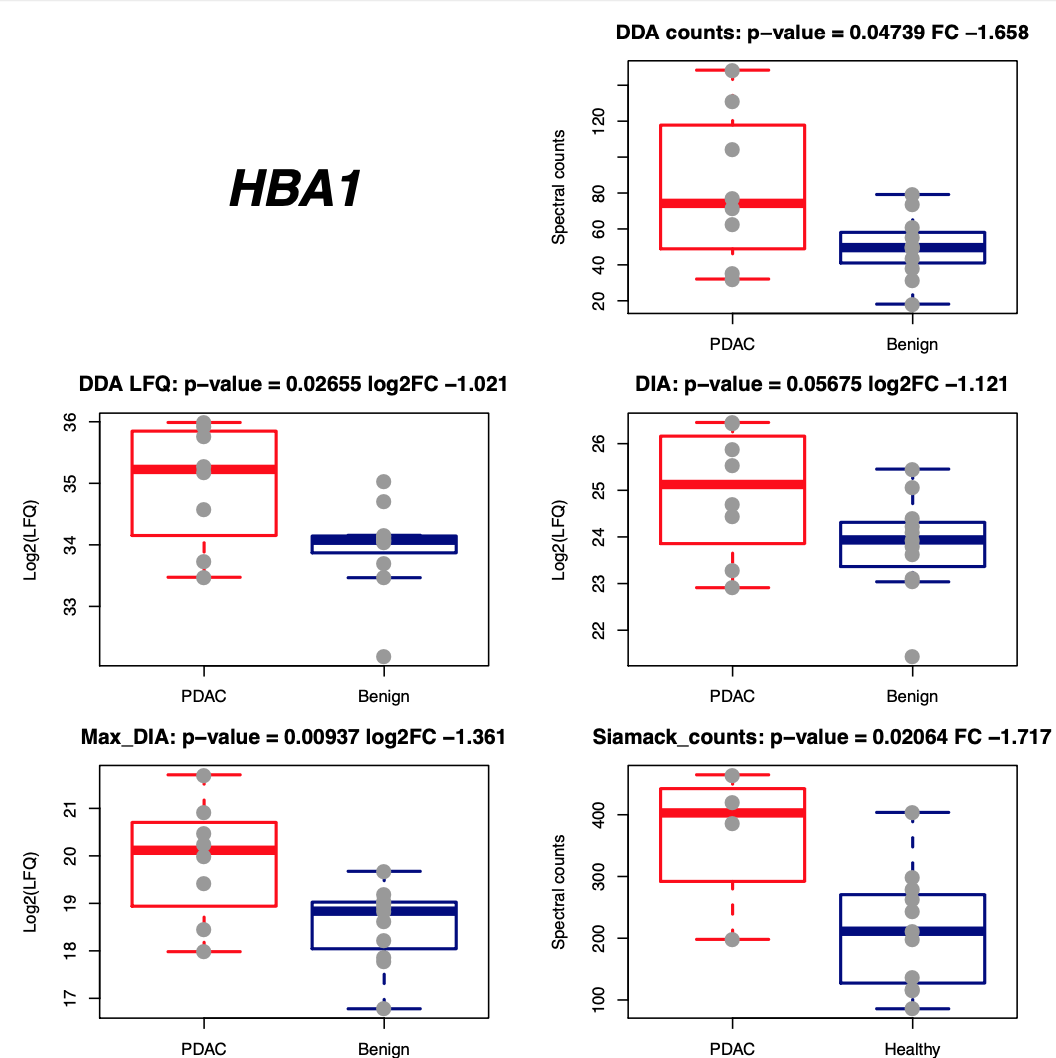


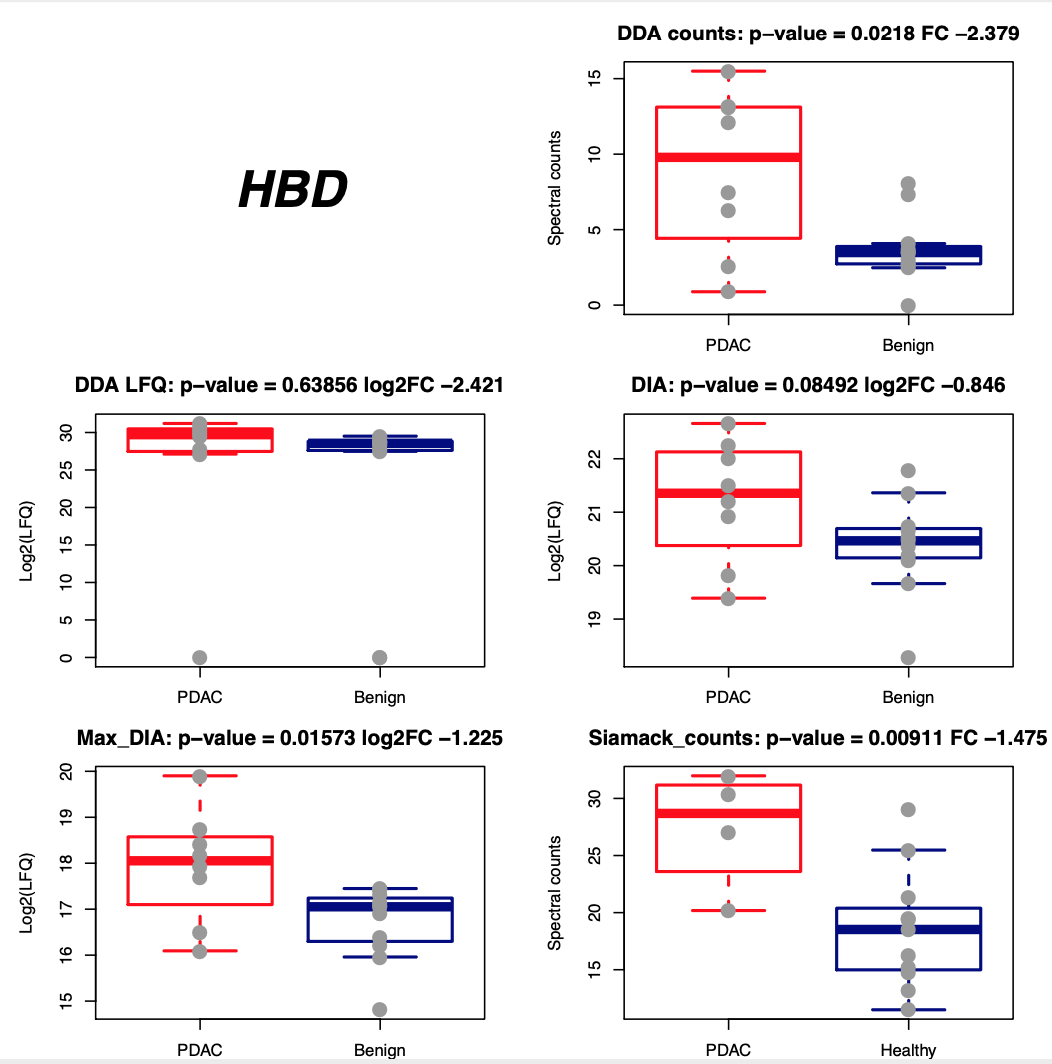


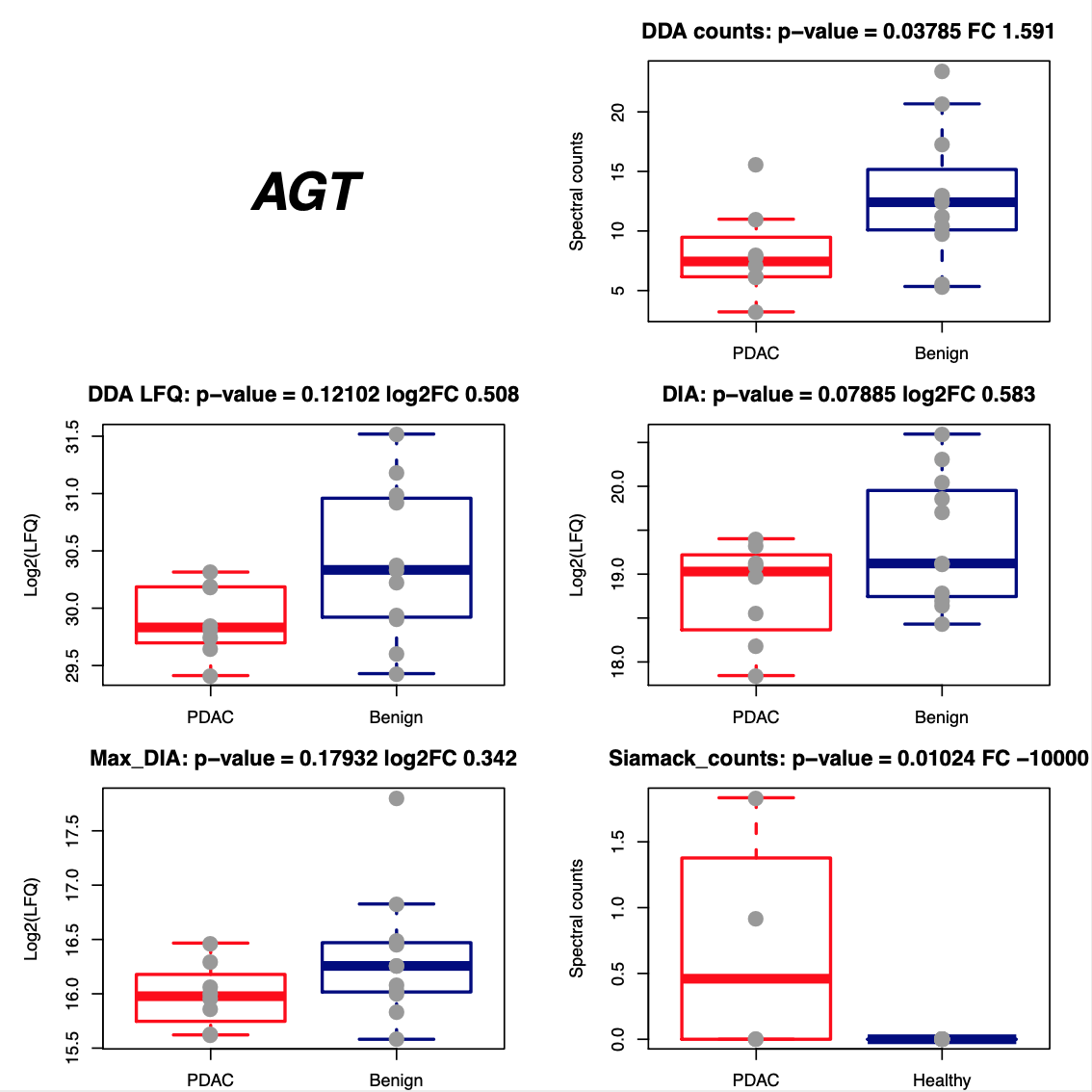


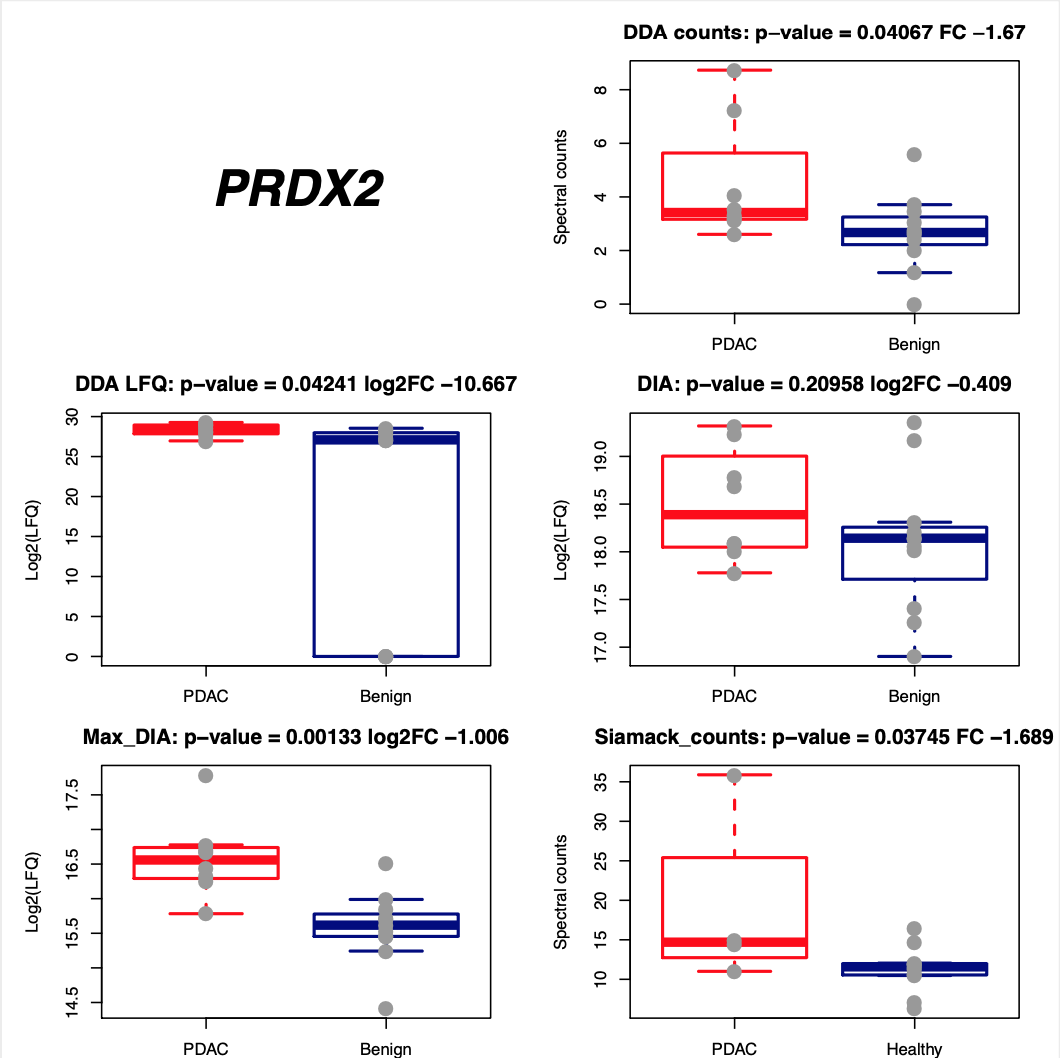


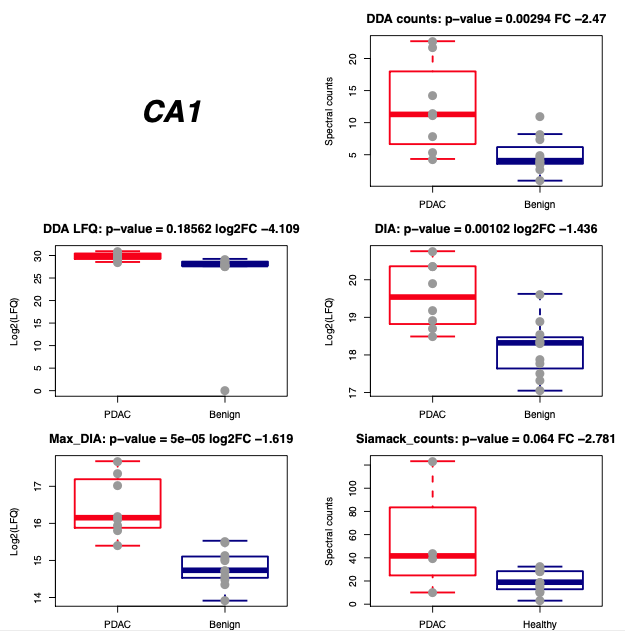

Supplement: Supplementary file 1 [file cancers-13-00066-s001.zip › suppl.Figures/FigureS6.docx]

## Transcriptomics (n=22)

sex F M

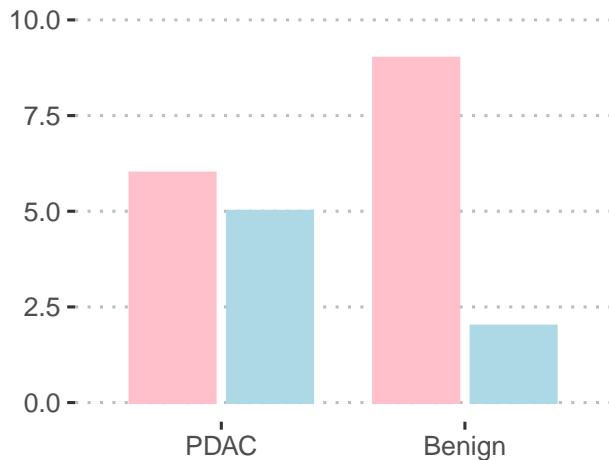

## Proteomics (n=12)

sex F M

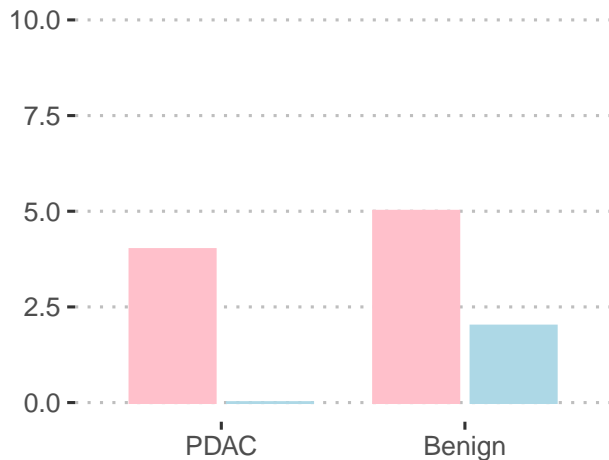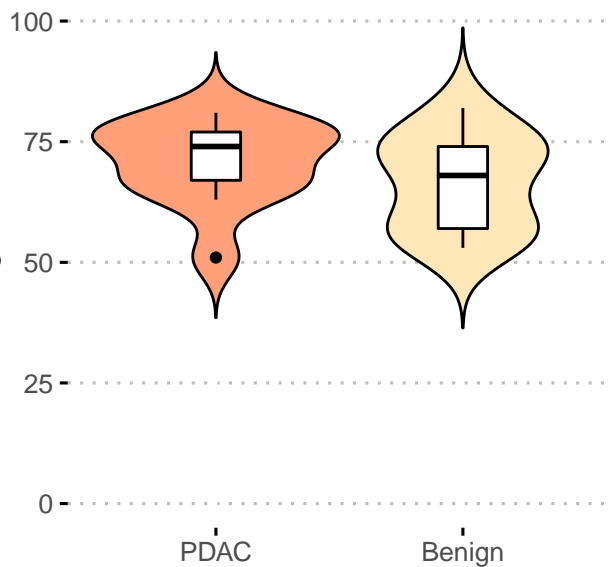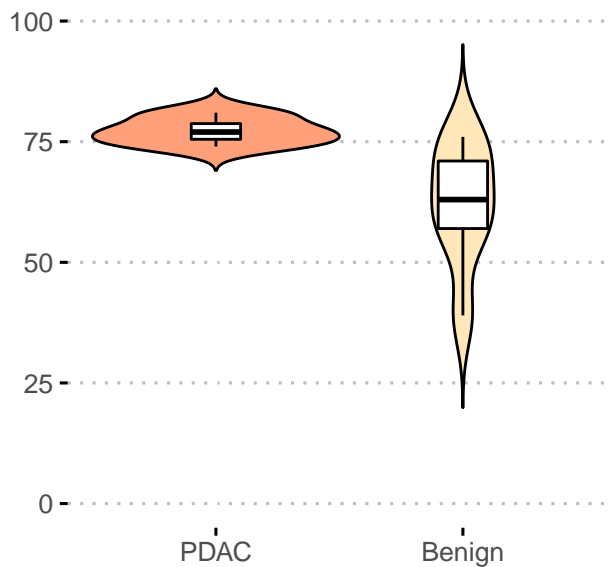

Supplement: Supplementary file 1 [file cancers-13-00066-s001.zip › suppl.Figures/FigureS2.pdf]

miRNA-22    + High    + Low

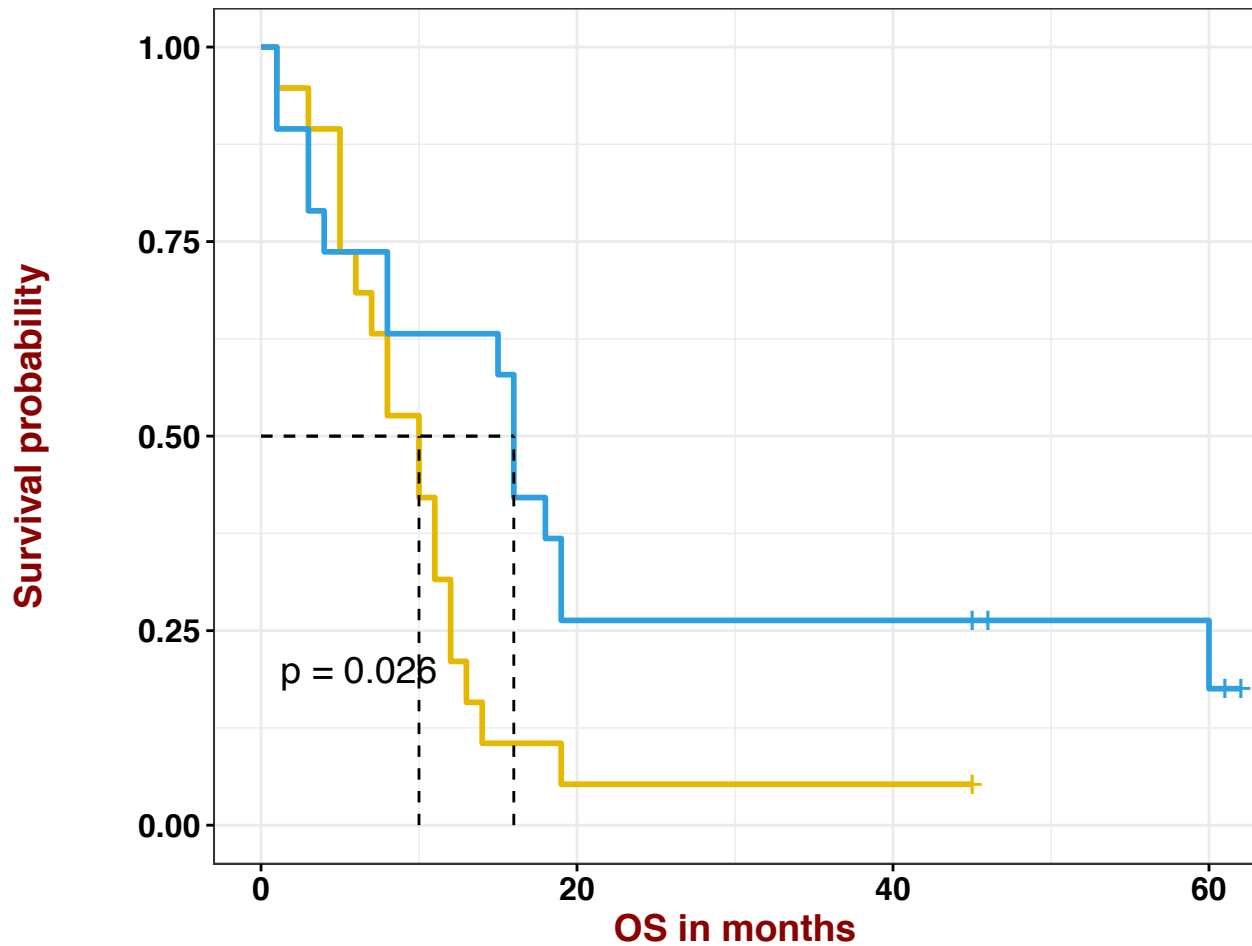

Number at risk

miRNA-22  
High  
Low

|    |   |   |   |
|----|---|---|---|
| 19 | 1 | 1 | 0 |
| 19 | 5 | 5 | 3 |

OS in months

Supplement: Supplementary file 1 [file cancers-13-00066-s001.zip › suppl.Figures/FigureS3.pdf]

**A**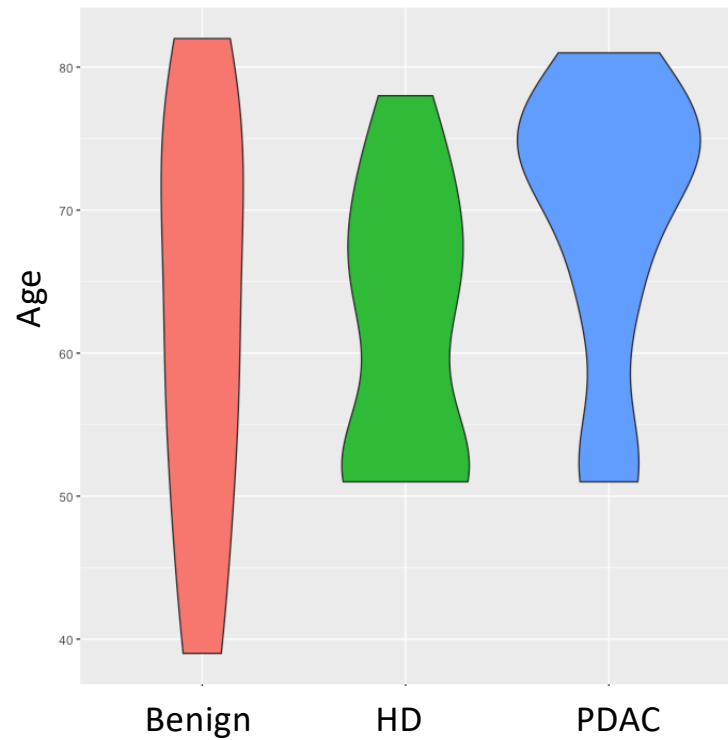**B**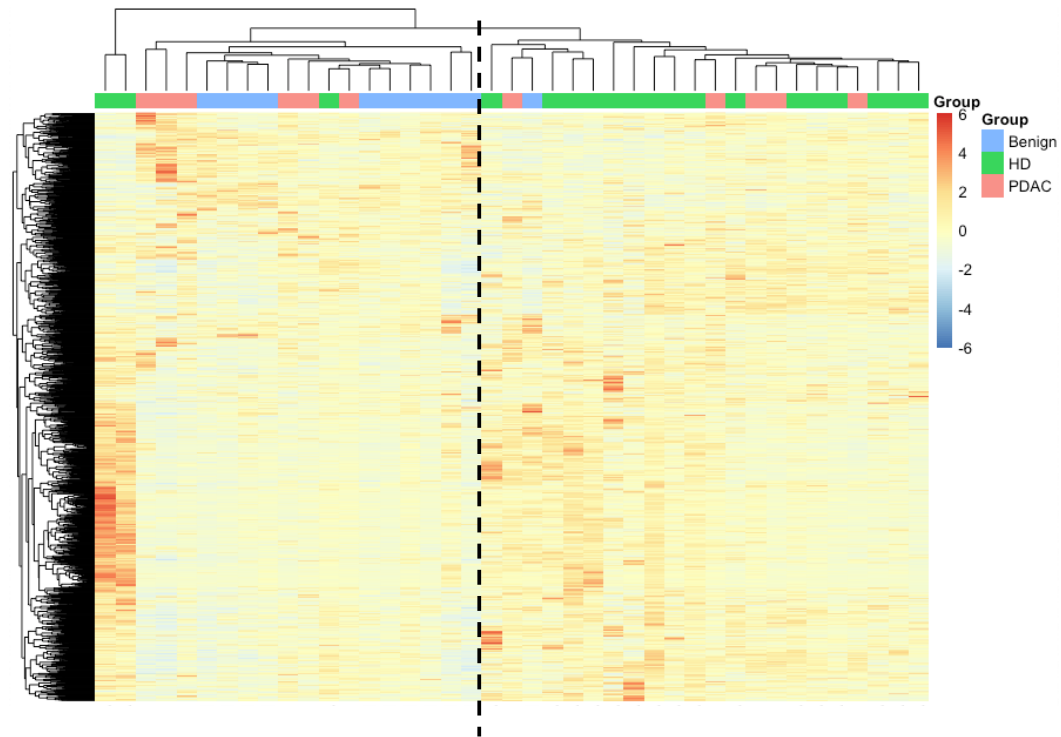

Supplement: Supplementary file 1 [file cancers-13-00066-s001.zip › suppl.Figures/FigureS1.pdf]

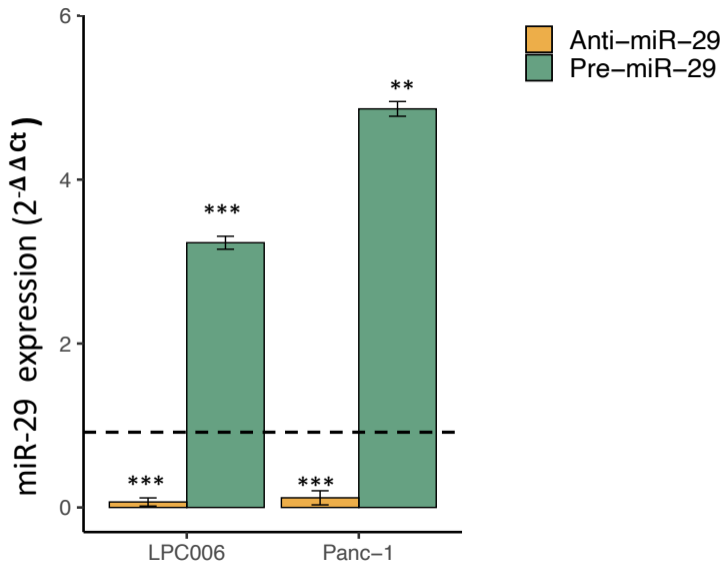

Supplement: Supplementary file 1 [file cancers-13-00066-s001.zip › suppl.Figures/FigureS5.pdf]
